# Multiple myeloma risk linked to DNA damage response genes

**DOI:** 10.1186/s13045-025-01776-1

**Published:** 2026-01-06

**Authors:** Michael Conry, Irina Ostrovnaya, Yelena Kemel, Saloni Sinha, Linda B. Baughn, Brian Avery, Kylee Maclachlan, Victoria Groner, Lauren Banaszak, Aaron Norman, Nicholas J. Boddicker, Alyssa Clay-Gilmour, Shaji Kumar, Ellen Kim, Sita Dandiker, Mitul Waghmare, Susan Slager, Douglas W. Sborov, Judy Garber, Elizabeth E. Brown, Michelle Hildebrandt, Parameshwaran Hari, Nicola Camp, Celine Vachon, Saad Usmani, Kenneth Offit, Vijai Joseph

**Affiliations:** 1 Clinical Genetics Service, Department of Medicine, Memorial Sloan Kettering Cancer Center, New York, NY USA; 2 Department of Epidemiology and Biostatistics, Memorial Sloan Kettering Cancer Center, New York, NY USA; 3 Department of Laboratory Medicine and Pathology, Mayo Clinic, Rochester, MN USA; 4 Department of Internal Medicine, Huntsman Cancer Institute University of Utah, Salt Lake City, UT USA; 5 Myeloma Service, Department of Medicine, Memorial Sloan Kettering Cancer Center, New York, NY USA; 6 Department of Quantitative Health Science Research, Mayo Clinic, Rochester, MN USA; 7 Department of Epidemiology & Biostatistics, Arnold School of Public Health, University of South Carolina, Columbia, SC USA; 8 Division of Hematology, Department of Medicine, Mayo Clinic, Rochester, MN USA; 9 Department of Medical Oncology, Dana-Farber Cancer Institute, Boston, MA USA; 10 Department of Pathology, The University of Alabama at Birmingham, Birmingham, AL USA; 11 Department of Lymphoma/Myeloma, MD Anderson Cancer Center, Houston, TX USA; 12 Department of Medicine, Weill Cornell Medical College, New York, NY USA; 13 Department of Medicine, Medical College of Wisconsin, Milwaukee, WI USA; 14 1275 York Ave, Box 295, New York, 10065 NY USA

**Keywords:** Cancer genetics, Cancer Prevention, Genetic Risk Factors, Germline Predisposition, Plasma Cell Neoplasms, Rare Pathogenic Variants, Cancer Predisposition Syndromes, Rare variants, Familial Hematologic Malignancies, Survival and Prognostic Biomarkers

## Abstract

**Background:**

DNA damage response genes (DDRG), implicated in several cancers as both predisposing risk factors as well as biomarkers for aggressiveness, have not been fully explored in multiple myeloma (MM).

**Methods:**

Herein, we analyzed disease associations of pathogenic variations in nine putative candidate genes using 3 446 MM cases and 323 233 cancer-free controls.

**Results:**

Increased MM risk was found to be associated with inherited rare pathogenic mutations in *TP53*,* ATM*,* CHEK2*,* KDM1A*,* and ARID1A*, with an enrichment of these variants among individuals with early onset or family history of MM. Individuals with *TP53* or *ATM* germline mutations are also likely to have worse overall survival.

**Conclusions:**

Our results suggest expansion of the phenotypic spectrum of some of these DDRG to include MM. The identification of these germline predisposition genes opens the avenue for targeted screening of higher risk individuals especially those with young-onset or a family history of plasma cell gammopathies.

**Supplementary Information:**

The online version contains supplementary material available at 10.1186/s13045-025-01776-1.

## Introduction

Multiple myeloma (MM) is a rare, but incurable blood cancer that arises from plasma cells in the bone marrow. Although genetic abnormalities - including chromosomal translocations such as t(11;14) and t(4;14), copy number aberrations and mutations in key driver genes such as *TP53*,* KRAS*,* NRAS*,* DIS3 -* are considered hallmark somatic events of these tumors [1, 2], relatively little is known about the germline components that may predispose individuals to increased risk of MM [3]. Several lines of evidence suggest a relevant role of heritable genetic factors in the germline that contributes to myelomagenesis. An increased risk of MM in family members compared with the general population has been shown by several studies [4]. MM or its precursor monoclonal gammopathy of undetermined significance (MGUS) has been shown to aggregate also in certain solid tumor families such as prostate cancer [5], suggesting shared susceptibility genetics. In recent years, association of DDRG to several cancer types has been established [6, 7]. Two recent studies [8, 9] have explored the hypothesis that rare, moderate to high penetrant pathogenic variants (germline mutations) in DNA damage response genes (DDRG) are causal in MM. Neither of these smaller studies explored the role of germline gene mutations in overall survival. Knowledge of DDRG status has enabled therapeutic interventions in solid tumors and it is reasonable to ask if those genes are relevant in MM [10] and if they are biomarkers of outcome. A broader understanding of the prevalence and effects sizes of risk of mutations in DDRG will define relevant etiologic pathways associated with MM onset and facilitate formulation of clinical approaches to targeted prevention, treatment, and surveillance plans.

We assembled a unique set of MM cases enriched for family history of MM/MGUS or lymphoma and/or with an earlier age at onset and compared the presence of pathogenic/likely pathogenic variants in the most likely candidate DDRG against a large, healthy, non-cancer controls in the UK Biobank [11]. For this study, we only selected five well known cancer predisposition genes (*ATM* [12], *BRCA1* [13], *BRCA2* [14], *CHEK2* [15], *and TP53* [16]*)* and four putative candidate MM risk genes from prior studies, namely *ARID1A* [17], *DIS3* [18], *KDM1A* [19], and *USP45* [17]. Our gene selection relies on two approaches. First, we wanted to determine if the most prolific pathogenic mutated genes in other cancers were relevant in MM. Genes routinely clinically tested in solid cancers such as *BRCA1/2*,* CHEK2*,* ATM* and *TP53* comprised this category. For our second approach specific to MM predisposition, we chose only those genes that were reported in the literature from smaller studies that utilized familial approaches and next generation sequencing. These yielded genes such as *KDM1A*,* USP45*,* ARID1A*,* DIS3*. We believe this represents a reasonable gene-set to explore the role of germline susceptibility genes in MM, in a rare variant context. A key factor in limiting our gene selection was evidence for reported variant pathogenicity by the community in the ClinVar database and prior literature [20], since genes with very few pathogenic variants were unlikely to yield evidence for an association. Most of these genes satisfied that requirement, although *ARID1A* was less informative. We did not include any genome-wide association study (GWAS) hits or genes near GWAS-identified loci. It is widely believed that GWAS variants act on non-coding regions using gene-regulation as its primary mechanism, whereas rare-variant association studies (RVAS) utilize well curated sets of pathogenic and likely pathogenic variants with evidence of disruption to protein-coding regions, resulting in a loss of function (LoF). Since MM has hardly any well-established predisposition genes, it seemed much prudent to focus on traditional genetic predisposition genes as well as previously implicated genes with coding LoF variations in our candidate genes selection.

## Methods

### MM cases

Germline DNA was collected from individuals with a confirmed diagnosis of MM from five centers (Supplemental Table 1, Supplemental Fig. 1). Cases were enriched for early onset (Supplemental Fig. 2) or a family history of MM. Genomic DNA of 2452 individuals with confirmed diagnosis of MM were collected through a consortium approach from Memorial Sloan Kettering Cancer Center, the Bone and Marrow Clinical Trial Network [21] (BMT-CTN0201), Mayo Clinic, University of Utah, University of Alabama at Birmingham, Dana Farber Cancer Institute, and MD Anderson Cancer Center, henceforth collectively described as MMSEQ. Where available, clinical information was also collected for each case, including age at diagnosis, stage at diagnosis according to ISS classification, hematologic indices at diagnosis, treatment type, and overall survival. The germline variation data for these MM cases were processed as described in the supplement. This research was approved by MSKCC institutional review board and all human participants gave written informed consent.

### Next generation sequencing

Genetic variations obtained from exome sequencing was generated as described in the supplement. Joint genotyping was performed using GATK (RRID: SCR_001876) version 3.7 to produce a multi-sample VCF according to the GATK best practices [22]. Quality control (QC) was applied to exclude variants with low genomic quality or samples that had high missingness. Variant records were annotated using CAVA2 [23] and Annovar [24] (RRID: SCR_012821) taking into account ClinVar (RRID: SCR_006169) assertions and SpliceAI [25] (RRID: SCR_026278) scores. The variant records were analyzed by PathoMAN [26] (RRID: SCR_026552), which asserts variant classification for germline variation as pathogenic, likely pathogenic (P/LP), variant of unknown significance (VUS), benign and likely benign (B/LB). Variants were curated as described in the supplement. Only P/LP variants qualified for further analysis and are found in the supplementary data file.

### UKB cases and controls

**Fig. 1:**
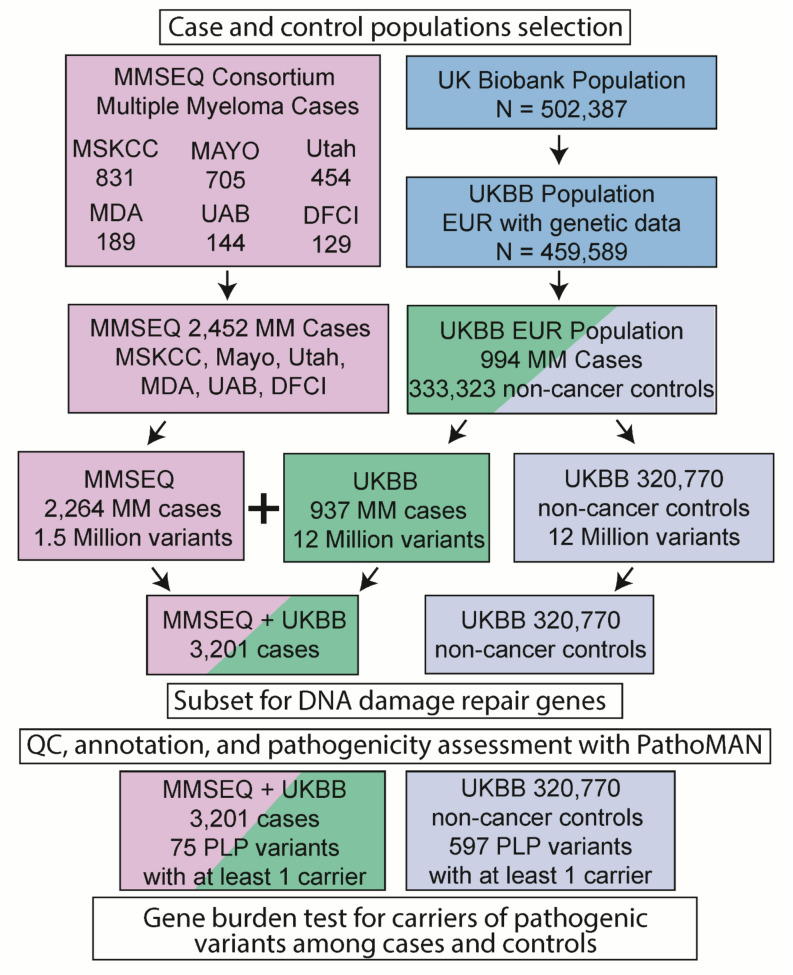
MMSEQ and UKBB population selection, QC, Annotation and analysis. Case-control population selection. MM cases were drawn from the MMSEQ consortium and UKB MM cases. Controls were derived from UKB non-cancer controls. See methods for detailed descriptions of population selection criteria, variant QC, annotations, pathogenicity assessment and statistical analysis

The UK Biobank (UKB) (RRID: SCR_012815) has collected genetic and health information from 502,387 on human health and diseases [11]. MM cases and non-cancer controls from the UKB were selected using criteria as described (Supplement). We identified and subset data from 937 prevalent and incident MM cases and 320,770 controls in this study from UKB (Fig. 1, Supplemental Table 2).

For the nine genes of interest, we used the DNAnexus (RRID: SCR_011884) research analysis platform to extract gene regions from UKB’s whole-exome sequence data. The GRCh38 coordinates for each gene were obtained from UCSC’s genome browser (RRID: SCR_005780). We divided the extracted exome VCF data into cases and controls before normalizing indels and converting multiallelic variants into separate records with bcftools (v.1.16) (RRID: SCR_005227). We then filtered the variants to retain only those with allele frequencies less than 1%. QC, annotation, and variant classification were performed the same as in the MMSEQ cases. We also note that both MMSEQ and UKB datasets were generated using the same sequencing platforms (NovaSeq 6000).

### Statistical analysis

The association between each candidate gene and MM risk was assessed by performing a gene-burden analysis in cases and controls using Fisher’s exact test with multiple testing correction using Benjamini-Hochberg method. Odds-ratios (OR) and 95% confidence intervals (95% CI) were calculated. Where available, clinical variables such as age at diagnosis, sex, and stage (ISS) were compared between carriers and non-carriers of each gene amongst cases to explore their role in genetic risk to MM subsets using the Wilcoxon rank sum test.

For each gene of interest, we performed a gene burden test by computing the cumulative allele count from MMSEQ and UK Biobank cases compared to UK Biobank controls. Using RStudio Version (2023.09.1) with R Version (4.2.2) (RRID: SCR_001905) we performed two-sided Fisher’s exact tests and accounted for false discovery rate owing to multiple testing problems by adjusting the p-values with Benjamini-Hochberg correction. We tested for the presence of major cytogenetic abnormalities [t(4;14), t(11;14), deletion of *TP53*] between carriers and non-carriers among cases (Supplemental Table 3).

**Fig. 2:**
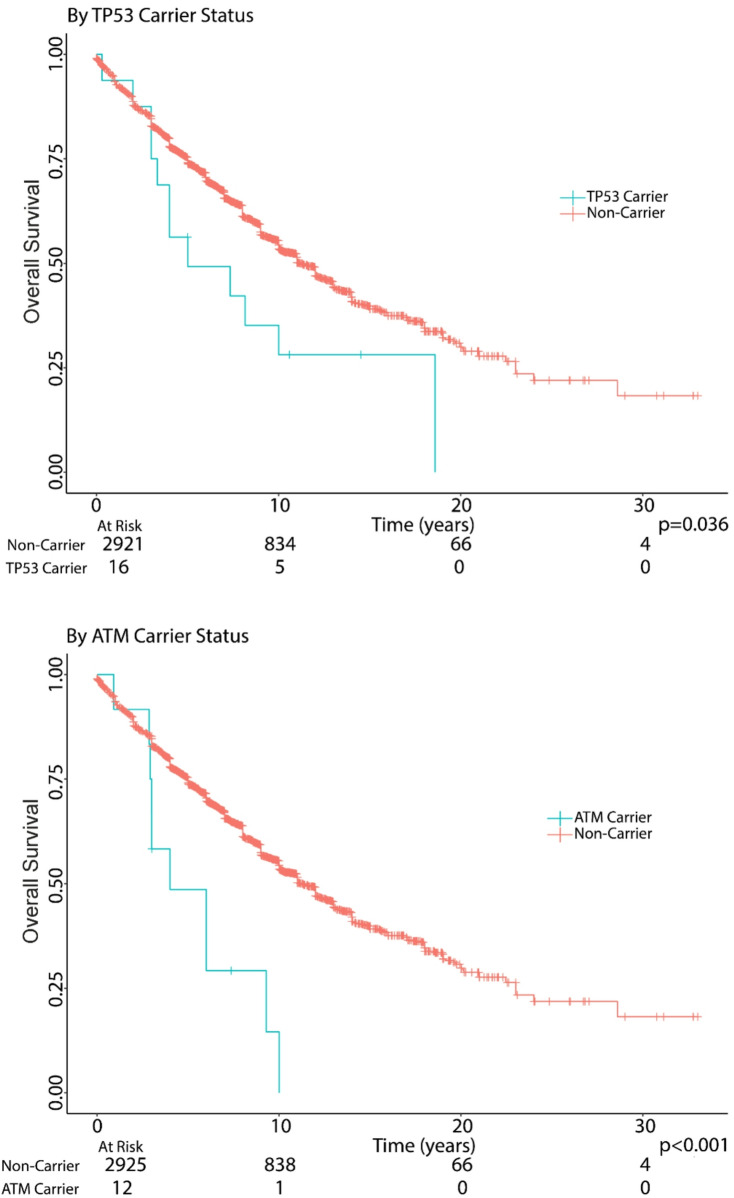
Overal survival by pathogenic carrier status in DNA damage response genes amoung MM cases. Overall survival (OS) by carrier status. The left panel shows Kaplan-Meier estimates of OS (in years) by *TP53* carriers to non-carriers. The right panel shows Kaplan-Meier estimates of OS by *ATM* carriers to non-carriers

Overall survival (OS) was analyzed using Kaplan-Meier curves and univariate and multivariate Cox proportional hazard regression. Using Cox regression analyses, we evaluated associations for individuals who carried a pathogenic variant in a specific gene with overall survival (OS). Models were adjusted for cohort (center), sex, and age at diagnosis. Among the 7 centers, there were 1306/2937 patients with death records available for the OS data. Since only 4 genes showed mutations in greater than 3% of cases, we limited OS analyses to just those genes (*TP53*,* CHEK2*,* DIS3*,* and ATM*). OS was defined as time from diagnosis to death or last follow-up. For 3 cohorts, Utah, MDA and UAB, we imputed the last year of follow-up to be the last year of death in that cohort. We could not obtain OS for BMT-CTN cohort since we had no follow-up information (Fig. 2). Cytogenetic data were included where available. Median follow-up time was estimated by the reverse Kaplan-Meier approach using the Survfit function in the R survival package (RRID: SCR_021137).

## Results

This study is comprised of 3446 MM cases and 333323 non-cancer controls (Table 1), of whom 3201 cases and 320770 controls passed QC. To our knowledge, this is the largest rare variant genetic variation study of MM patients. Thibaud et al. [9], recently, used the CoMMpass and ISMMS populations of *N* = 895 and *N* = 786, respectively, while Boddicker et al. [8] used the Mayo Clinic cohort of 2138 MM cases and 42,632 controls. Two separate ascertainments, MMSEQ (*n* = 2264) and UBB-Cases (*n* = 937) contributed to the 3201 cases (Table 1**)**. The mean age at MM onset of our MMSEQ cases is 55.2 while the mean age for UKB cases is 66.4. Controls were only sourced from the UKB. Details of each of the case and control series are provided in Table 1 and Supplementary methods. We found that 4.6% of MM cases carried qualifying variants in these nine genes compared to 1.5% of controls. Pathogenic variants were observed 3 times more among cases than in controls. The results **(**Table 2**)** show that *CHEK2* (3.86 [95%CI: 3.1–4.8], *p* = 7.20 × 10^− 23^), *TP53* (45.05 [95%CI: 23.8–81.8], *p* = 1.68 × 10^− 20^), *ATM* (3.55 [95%CI: 2–5.8.8], *p* = 7.39 × 10^− 5^) and *KDM1A* (4.88 [95% CI: 1.9–10.3], *p* = 1.48 × 10^− 3^) were significantly associated with the risk of MM, adjusted for multiple testing correction (Benjamini-Hochberg). *ARID1A* was also significantly associated despite carrier counts being low at 3 carriers (0.09%) from cases and 8 carriers (0.002%) from controls (37.6 [95% CI: 6.4–157.1.4.1), *p* = 3.37 × 10^− 4^). While there was a preponderance of males in the MM cases and a greater number of females in controls, the same 5 genes remain significant even after adjusting for sex using a Mantel-Haenszel test (Table 2).

**Table 1:** Demographic data for MM cases and non-cancer controls

Characteristic	Overall *N*=323,971		Cases *N*=3201		Controls *N*=320,770	
**Age**	Combined Ages		Age at Diagnosis		Age at Recruitment	
<40	115	<0.1%	110	3.40%	5	<0.1%
40–45	37,682	11.60%	171	5.30%	37,511	11.70%
46–50	48,398	14.90%	360	11.20%	48,038	15.00%
51–55	54,454	16.80%	567	17.70%	53,887	16.80%
56–60	64,250	19.80%	699	21.80%	63,551	19.80%
61–65	70,779	21.80%	675	21.10%	70,104	21.90%
>65	39,189	12.10%	619	19.30%	38,570	12.00%
**Sex**						
Female	175,077	53.60%	1298	40.50%	173,779	54.20%
Male	148,894	45.40%	1903	59.50%	146,991	45.80%
**Ethnicity**						
European	323,917	99.90%	3147	98.30%	320,770	100%
African American	5	<0.01%	5	0.20%	0	0%
East Asian	4	<0.01%	4	0.10%	0	0%
Native American	4	<0.01%	4	0.10%	0	0%
Hispanic	2	<0.01%	2	0.10%	0	0%
Other	2	<0.01%	2	0.10%	0	0%
Unknown	37	0.01%	37	1.20%	0	0%
**Vital Status**						
Alive	310,970	95.90%	1751	54.70%	309,219	96.40%
Deceased	12,884	3.97%	1333	41.60%	11,551	3.60%
Lost to follow-up	5	<0.01%	5	0.20%	0	0%
Unknown	112	0.04%	112	3.50%	0	0%

The table describes the demographic data for the MMSEQ population, UK Biobank cases and controls, split by age at diagnosis, sex, ethnicity, and vital status. UK Biobank vital status was reported from the death registry and may not be complete for all participants

**Table 2:** Gene Burden Test Results for each

GENE	Cases (%)	Controls (%)	*p*-value	Odds Ratio	MH_OR	MH_p-value
*CHEK2*	85 (2.66)	2250 (0.70)	**7.20E-23**	3.86 [3.1–4.8]	3.85	4.28E-37
*TP53*	17 (0.53)	38 (0.01)	**1.68E-20**	45.05 [23.8–81.8]	44.05	3.40E-99
*ATM*	16 (0.49)	453 (0.14)	**7.39E-05**	3.55 [2–5.8.8]	3.09	6.52E-05
*ARID1A*	3 (0.09)	8 (0.002)	**3.37E-04**	37.61 [6.4–157.3.4.3]	39.38	8.43E-13
*KDM1A*	7 (0.21)	144 (0.04)	**1.48E-03**	4.88 [1.9–10.3]	4.91	6.52E-05
*BRCA1*	4 (0.12)	138 (0.04)	7.97E-02	2.9 [0.78–7.62]	2.55	1.63E-01
*DIS3*	11 (0.34)	706 (0.22)	1.58E-01	1.56 [0.78–2.82]	0.17	1.08E-01
*USP45*	1 (0.03)	429 (0.13)	1.58E-01	0.23 [0.006–1.3.006.3]	1.58	2.06E-01
*BRCA2*	9 (0.28)	574 (0.17)	2.00E-01	1.57 [0.71–3.01]	1.48	3.32E-01

The table shows the results of the gene burden analysis and statistics from fisher’s exact tests. The cases and controls columns are counts of individuals found to be harboring a qualifying pathogenic or deleterious variant in a specific gene. Bolded p-values are significant. Sex-stratified odds ratios and p-values are derived from the Mantel-Haenszel test. P-values were adjusted using the Benjamini-Hochberg method

The overall carrier rate among MMSEQ cases was 5.6%, nearly twice the overall carrier rate of UKB cases at 2.9%. Gene-wise carrier rates were similar across case datasets with the notable exception of *TP53*,* BRCA2* and *KDM1A*. The *TP53* carrier rate was 0.7% in the MMSEQ cases, who were selected for early onset MM or a history of familial cancer, while only 0.1% in the UKB MM cases. Conversely, *BRCA2* carriers were underrepresented in the MMSEQ cases with a carrier rate of 0.2% compared to UKB case carrier rate of 0.4%. *KDM1A* MMSEQ carrier rate was 0.3% but no PLP variants were found in UKB cases.

Of 3 201 cases, 2937 were included in OS analysis with 264 removed owing to missing information. Of those, 1 306 died, and median follow up time was 10 years. We found that MM patients who carried *TP53* or *ATM* mutations had worse survival compared to wild type (Fig. 2). Specifically, *TP*53 carriers had significantly poorer OS compared to non-carriers of *TP53* (HR = 1.9 [95% CI: 1.06–3.3], *p* = 0.031). The association remained significant after adjusting for cohort (center), gender and age (modeled as cubic spline): (HR = 1.92, 95% CI 1.1–3.4, *p* = 0.025). Of the 17 *TP53* carriers, 6 had cytogenetics reported and one had a reported somatic *TP53* loss. Similarly, *ATM* carriers had worse OS (HR = 2.9, [95% CI: 1.5–5.4], *p* = 0.0009) than non-carriers of *ATM*, and remained significant after adjusting for cohort, gender, and age: (HR = 3.09, 95% CI 1.7–5.8, *p* = 0.0004). CHEK2, despite having the largest number of carriers (*n* = 85), shows no significant survival difference (*p* = 0.9), suggesting that germline CHEK2 variants primarily influence disease susceptibility rather than outcome (Supplementary Fig. 6).

**Fig. 3:**
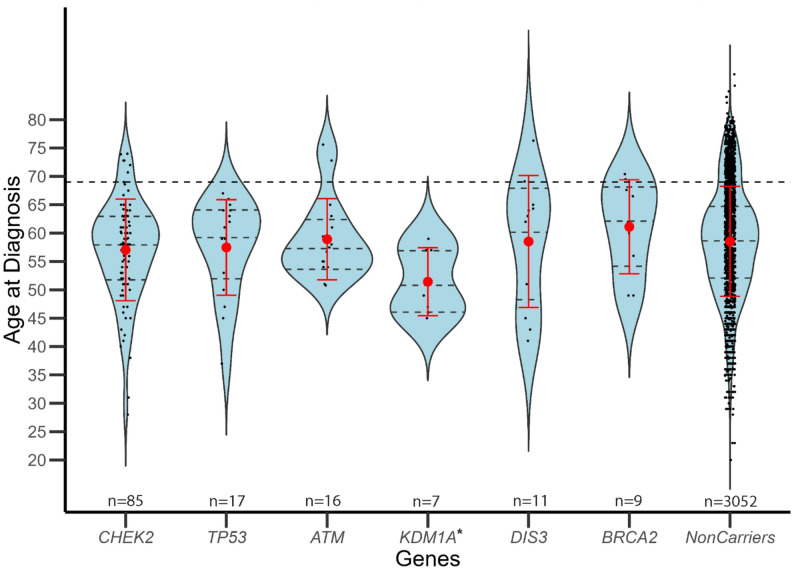
Distribution of age at diagnosis amoung carrires of cancer predisposition genes. Age at diagnosis by carriers of gene-specific pathogenic variants. The age at diagnosis of carriers of pathogenic or deleterious variants is distributed younger than the average age of diagnosis for multiple myeloma. The interquartile range is shown as a dashed line within the blue shaded region of the violin plot. The central dashed line within each plot is the median age and the mean and standard deviation is shown in red. The horizontal line denotes the median age at onset (69 from SEER) of multiple myeloma. The * indicates that the age at diagnosis for carriers of *KDM1A* is significantly distributed younger compared to the non-carriers. Genes with 4 or fewer pathogenic variants among carriers are not displayed

**Fig. 4:**
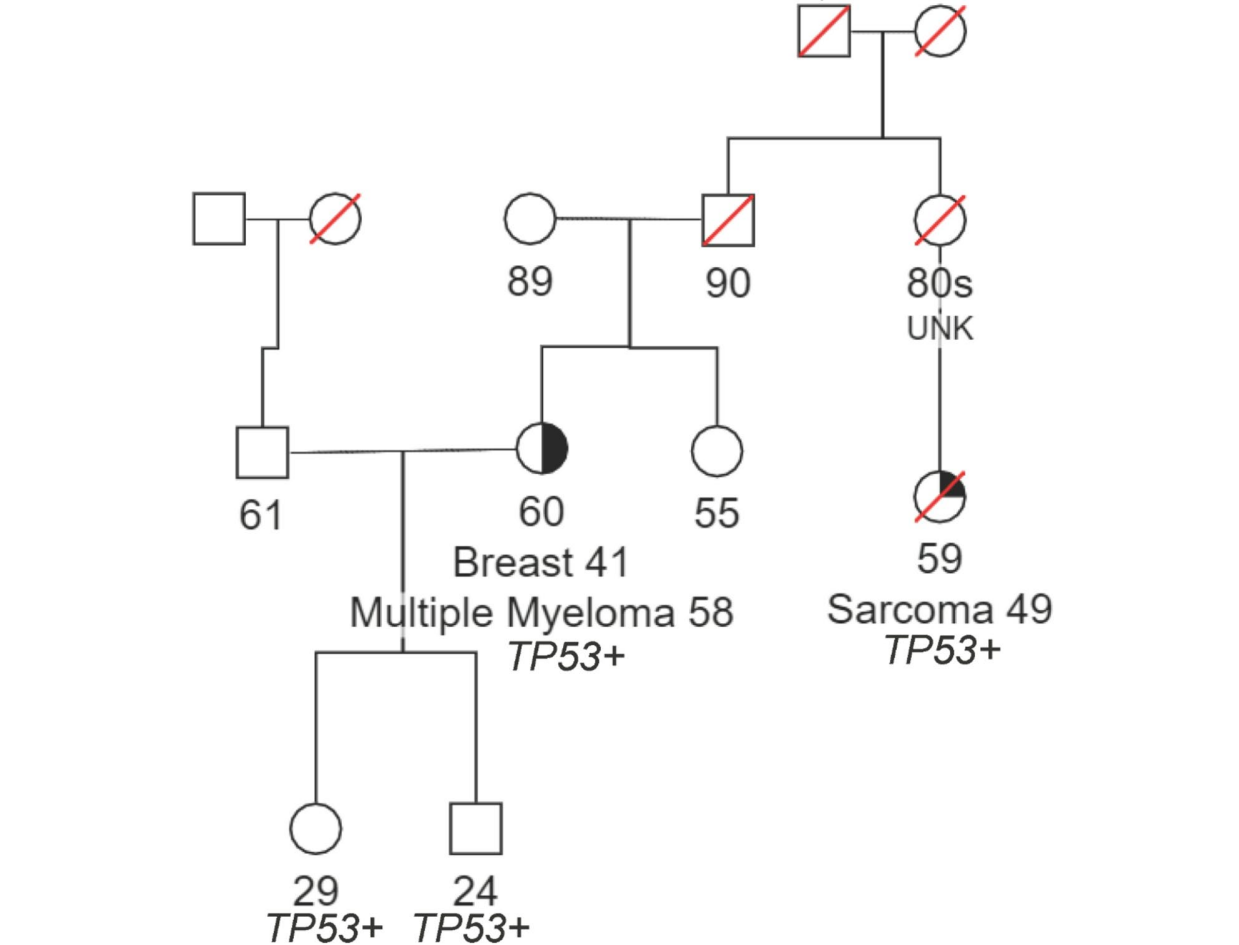
Pedigree from a MM proband with pathogenic *TP53* mutations amoung family members. A pedigree from a MM proband with pathogenic TP53 mutations among close relatives. The pedigree shows a shared *TP53* pathogenic mutation among first- and third-degree relatives. The cousin developed sarcoma in their late 40 s which is common among those with *TP53* mutations. We hereby confirm that the consent of the relevant patient(s) has been obtained for inclusion of MM pedigree

Among the 148 mutation carriers, the lowest median age at diagnosis was for *KDM1A* at 49 years (range 45 to 59) and the highest was for *DIS3* at 62 years (range 37 to 67 years), significantly younger than the median age at diagnosis of MM (69 years) in SEER cancer stats. We found *KDM1A* (*n* = 7) to have a younger age of onset by carrier status (*n* = 7, *p* = 0.026). (Supplemental Table 4, Fig. 3). Univariate analysis of age at diagnosis, ISS stage, along with measurements of free kappa, free lambda, kappa-lambda ratio, beta-2-microglobulin, albumin, creatinine, calcium, plasma cells, hemoglobin, and serum M-spike levels between carriers and non-carrier showed no significant differences. Cytogenetic abnormalities associated with a high-risk MM diagnosis (loss of *TP53*, t(4;14), or t(11;14)) were available among 27% (*n* = 620) of the cases in MMSEQ. Analyses of these common cytogenetic abnormalities among carriers from MSKCC did not show an association with DDRG mutations (Supplemental Table 3). However, three individuals who harbored a *TP53* pathogenic variant also showed evidence of somatic loss of *TP53* in the tumor. Of the 17 TP53 mutation carriers identified across MMSEQ and UKB, 10 had available cytogenetic information, of which 30% showed TP53 loss. Among cases with available ISS stage at diagnosis (*N* = 1360), we did not find a significant difference (Wilcoxon ranked-sum test *p* = 0.876) in stage at diagnosis between carriers (*N* = 86) and non-carriers (*N* = 1274) (Supplemental Fig. 4). No co-segregation of DDRG mutations was found among the few MM-affected first- or second-degree relatives of the probands studied with WES available (3 kindreds, 8 individuals). One kindred with a germline *TP53* mutation and history of early-onset MM, breast cancer and sarcoma were reported among the families with a history of MM seen at the MSKCC CGS, suggesting MM could be part of the cancer spectrum in Li-Fraumeni syndrome (LFS). (Fig. 4).

## Discussion

The identification of germline predisposition variants in MM is a first step towards understanding the roles of heritable genetic risk and how they lead to oncogenic priming. Several of the genes studied here are established, inherited markers of *bona fide* cancer syndromes. Our results show compelling evidence of *CHEK2*,* TP53*,* ATM*,* ARID1A* and *KDM1A* are associated with MM, which is novel. In addition, we examine and show how specific DDRG such as *TP53* and *ATM* also influence disease progression and survival. There are several unique characteristics of this study compared to recently published studies (Supplemental Table 6). In general, the MM cases here have a younger age at onset. The mean age at onset of MM of combined MMSEQ and UKB cases are a decade earlier (58.5 mean ± 9.7 SD) than the SEER age at diagnosis (69 years). Among MMRF CoMMpass patients reported in Thibaud et al. [9],, and Martins Rodrigues et al. [27],, the mean age at diagnosis is 62. Individuals whose age at diagnosis of MM was younger than 55 years accounted for 47% of the MMSEQ cohort and 16% in UKB. The MMSEQ cohort consists of familial cases with a family history of MM, MGUS, or lymphoma in first- or second-degree kinship (10.5%), increasing the chances of a heritable etiology of the disease [28]. These DDRG pathogenic variations in MMSEQ and UKB are being reported for the first time. They complement the MMRF cohort that has been used to study germline susceptibility in two recent reports [9, 27]. The MMRF cohort had a carrier rate of only 1.5% for the genes reported in this study, suggesting it lacks the loss of function enrichment and power to discover associations in *TP53*, *ATM*, or *CHEK2* compared to an early-onset/familial cohort. Among gene-specific findings, *KDM1A* carriers exhibited significantly younger age at diagnosis, consistent with its discovery in early-onset familial MM. This likely reflects *KDM1A* tumor suppressor role in regulating germinal center B-cell differentiation, where loss-of-function accelerates plasma cell expansion and shortens MGUS-to-MM latency. This finding supports targeted *KDM1A* testing in young-onset MM patients and their families.

A recent RVAS study from Boddicker et al. [8] of 2138 MM cases and 42632 controls found associations between carriers of pathogenic variants in *CHEK2 (n = 39*,* 1.8%)* and *TP53* (*n* = 5, 0.2%) and an increased risk of developing MM. Their study also suggested an association with *ATM carriers (n = 18*,* 0.8%)*; however, this association did not remain significant after correcting for multiple testing.

To our knowledge, our study is the only one that has showed a significant association between germline *ATM*,*TP53* mutations and MM etiology (Supplemental Table 6) as well as patients’ overall survival. *ATM* and *TP53* carriers show a 2 and 3-fold higher hazard of death compared to non-carriers, respectively. We found three more individuals in the *TP53* database (https://tp53.cancer.gov/) [29] with MM (Supplemental Table 5). *TP53* mutation carriers with MM were present, but not associated in both the familial and MMRF subsets of the study by Rodrigues et al. [27], as well as in another study of prostate cancer [30]. Additionally, emerging evidence suggests that germline *BRCA2* mutations, long associated with breast and ovarian cancers, may also predispose individuals to MM, although findings remain inconsistent across studies [27, 31]. We note the discordance in the above two reports, with the former showing a marginal association and the latter, showing a lack of association, highlighting larger sample size requirements for such a study. In this study we discovered twice the number of *BRCA2* carriers than previously reported, however failed to replicate these prior associations. An older study from the United Kingdom [31] of 513 MM cases and 1569 controls, as well as another European familial study [32] of 21 families both failed to identify any significant rare variant associations with MM. These findings underscore the need for diverse and well-characterized cohorts to elucidate the genetic architecture of MM. Our study has a multi-institutional cohort comprised of familial and early-onset cases and well as a population-based cohort from the UKB. Our controls are ancestry matched. The curation of pathogenic variations in these genes plays a critical role in gene-burden testing. However, there are inconsistencies in how variant curation has been performed in such studies. For example, Rodrigues *et al.* [27]., used three categories (P, LP, PVUS) that contributed variants that could affect risk, while Thibaud et al. [9], used two sets of criteria, one for autosomal dominant and other for (PGV-A, PGV-B/C) recessive and founder mutations. The study by Boddicker *et al.* used a similar curation process to ours and defined pathogenic variations to include loss-of-function variants or variants identified as P/LP in ClinVar. However, they used the Biological Reference Repository for annotation while we used PathoMAN [26] for curation. PathoMAN is a highly refined variant pathogenicity assertion algorithm that uniformly and consistently identified P, LP, VUS variants within both the cases and controls. Only P/LP were used as qualifying variants in further analysis.

Heterozygous carriers of *ATM* pathogenic variants have been found to be at increased risk for breast, pancreatic, and prostate cancers and more recently there have been suggested associations with ovarian cancer and some reports of lymphoid malignancies [33]. For example, *ATM*, in recessive forms, is associated with hematologic malignancy in children affected with ataxia telangiectasia [12, 15]. *CHEK2* was originally described as a variant of Li-Fraumeni syndrome [34], but has been associated with several cancer types [34, 35] including other lymphoid malignancies [36]. Here, these genes provide evidence for strong associations to MM, and further expand the phenotype to include MM, where DDRG play a pivotal role in cancer risk. Identification of family members of MM probands could benefit from emerging strategies to clinically screen for early detection of LFS.

This study identified five novel DDRG associations to MM risk in the germline by analyzing rare exonic P/LP variations (Supplemental Fig. 5). Our data also suggest that, these germline *TP53* mutations also lead to poor survival; similar to what has been observed in somatic *TP53* loss [37]. Notably, our analysis did not identify any *TP53* MM proband meeting Chompret criteria (based on family history) for clinical diagnosis of Li-Fraumeni Syndrome (LFS). Because our analysis was not performed in a clinical context, however, it is possible that more detailed review of family history might have revealed syndromic features associated with lower penetrance. *TP53* mutations were described previously in families with MM as a cancer type within LFS (Fig. 4) [30, 38]. We found three more reported MM cases in the *TP53* database (Supplemental Table 5), suggesting MM may be a component of LFS, although further functional studies may be required to confirm causality.

Germline rare variants in exonic regions of cancer susceptibility genes usually form a long tail distribution. Our strategy here limited the search to a set of nine genes we believed would be represented sufficiently for a statistical association test. Despite the strong and modest associations that we discovered, it is evident that for an agnostic gene discovery of MM specific germline risk genes in the coding genes, a substantially larger number of cases is required for success. We are currently expanding our consortium to include additional sequenced MM cases, which will enable an adequately powered exome-wide rare variant analysis for novel gene discovery as a future publication. Methods such as meta-analyses of existing RVAS datasets would be a good starting point. To further discover novel cancer germline susceptibility genes with large effect sizes that can influence risk management in a clinical setting sample from more cohorts will need to be carefully selected and sequenced. An inherent issue with studying cancers with late onset and high lethality is that familial studies are usually limited to just the DNA of the proband and siblings and rarely are informative across generations [32]. Studies, including ours, are often enriched for individuals of European ancestry, thus preventing generalization of findings to other genetic ancestries. Although we know that African American populations have higher incidences and their risk is greater for MM [39], no large-scale studies have been conducted to understand the effects of rare DDRG mutations in that population or in other populations of the world [40]. Our study did not perform tumor normal sequencing across the cohort and hence is uninformative for Knudson’s two-hit theory in the tumor, where germline mutations were involved. Recent whole-genome sequencing of LFS tumors demonstrates that germline *TP53* mutations undergo copy number gain years before tumor diagnosis, with somatic alterations additionally affecting Wnt, PI3K/AKT signaling, and homologous recombination pathways [41]. Future studies integrating germline and longitudinal tumor sequencing will be essential to comprehensively characterize how inherited DDR defects contribute to MM pathogenesis. This study does not address risk factors such as obesity and diabetes.

Almost all cases of myeloma are preceded by a precursor disease state, MGUS, without target organ damage or clinical symptoms. Although the feasibility of intervention in the precursor phase is currently debated, studies (iSTOPMM [42], PROMISE [43]) that explore population screening approaches in germline findings identified here could allow selection of individuals with a strong family history or germline risk for targeted screening [44]. The value of early detection itself might also be different in incidentally detected asymptomatic gammopathy versus those with a germline risk identified through targeted screening [45]. More detailed clinical annotation of kindreds affected by these novel putative MM predispositions genes will allow designing of further studies to target screening and preventive strategies in high-risk populations.

## Conclusions

In our results, we have documented that, 5.6% of MMSEQ and 2.9% of UKBB MM cases had a pathogenic germline mutation in one of these nine genes. Our cohort’s median age at diagnosis was 58.5 years, notably younger than the CoMMpass study (63 years) and substantially younger than the SEER-reported population median of 69 years, reflecting our enrichment strategy for early-onset disease. This selection approach likely contributes to our observed 5% germline variant carrier frequency, which should be interpreted as an upper bound compared to unselected MM populations. Our data have numerous immediate clinical implications. Firstly, the yield of germline genetic testing in this cohort, 5%, is comparable to cancer types in which genetic testing is standard of care. For example, NCCN guidelines recommend universal germline genetic sequencing of patients diagnosed with exocrine pancreatic cancer, in which the yield of genetic testing is approximately 4–5% in unselected cohorts. Our data raises the question of whether universal germline genetic sequencing should be performed for all MM patients, similar to other hematologic malignancy subtypes. Secondly, the germline predisposition genes evaluated in this study are associated with other cancer risks, and detection of pathogenic variants may render patients eligible for surveillance procedures that have been shown to improve overall survival, such as whole-body MRI for LFS. In the current era, in which patients with MM have multiple treatment options and median overall survival exceeding a decade in many cases, it is imperative that patients continue to undergo age-appropriate and high-risk cancer screening if indicated based on germline status. Finally, detection of a pathogenic variant would allow for cascade genetic testing of family members, thus identifying unaffected individuals who would benefit from surveillance and risk-reducing interventions. Given that almost all cases of myeloma are preceded by a precursor disease state (MGUS), this high-risk cohort also represents the ideal population for studying the feasibility and efficacy of screening serum electrophoresis to allow for early detection and intervention. Intervention in the MGUS precursor phase is currently debated, but the value of early detection may be greater in those with an underlying germline risk identified by targeted sequencing. While this approach may not improve overall survival, early intervention may reduce the risk of MM-associated morbidity, such as end-stage renal disease or painful lytic bone lesions.

## Supplementary Information

Supplementary Material 1

Supplementary Material 2

## Data Availability

De-identified data will be available on request from [josephv@mskcc.org](mailto: josephv@mskcc.org) Data is deposited at dbGaP (Study accession id: phs002497.v1.p1).
