# Supplementary material for: Multiple myeloma risk linked to DNA damage response genes
Source: J Hematol Oncol. 2026 Jan 6;19:10. doi: 10.1186/s13045-025-01776-1 (PMC12810017; doi:10.1186/s13045-025-01776-1)
Supplement: Supplementary file 2 — Supplementary Material 2 [file 13045_2025_1776_MOESM2_ESM.docx]

# Supplementary Information

*List of tables and figures*

Supplemental Table 1 . . . . . . . . . . . . . . . . 6

Supplemental Table 2 . . . . . . . . . . . . . . . . 7

Supplemental Table 3 . . . . . . . . . . . . . . . . 8

Supplemental Table 4 . . . . . . . . . . . . . . . . 9

Supplemental Table 5 . . . . . . . . . . . . . . . . 10

Supplemental Table 6 . . . . . . . . . . . . . . . . 11

Supplemental Figure 1 . . . . . . . . . . . . . . . . 12

Supplemental Figure 2 . . . . . . . . . . . . . . . . 13

Supplemental Figure 3 . . . . . . . . . . . . . . . . 14

Supplemental Figure 4 . . . . . . . . . . . . . . . . 15

Supplemental Figure 5 . . . . . . . . . . . . . . . . 16

Sample Collections

Sample data was collected from 6 centers (MSK, UAB, MAYO, Utah, MDA, DFCI) along with clinical information. The clinical information was complete for some variables but sparse for others (Supplemental Table 1, Supplemental Figure 1, Supplemental Figure 2). Inclusion criteria for cases were as follows:

The inclusion criteria are anyone with MM, but the preferred criteria are:

Age of Onset <60, European

Family History of MM in first degree relative (FDR), second degree relative (SDR) or a family history of lymphoma in FDR/SDR (no age at diagnosis limit for the proband for familial samples).

Availability of 500ng of sequencing quality DNA

Institutional certification for data sharing

Completion of core variables and auxiliary variables.

a. Core Variables: mean age, sex, age at diagnosis of MM, ethnicity, and vital status.

b. Auxiliary Variables: history of heme malignancy in FDR, SDR, ISS stage, light chain, ploidy, cytogenetics, and date at last follow up.

Details of each of the participating study is given below:

Mayo Clinic study:

Ascertainment of MM cases and genotyping was approved by the Mayo Clinic IRB. The study included incident MM cases seen in the regional practice between 1998 and 2007 and recruited within 6 months of initial diagnosis. A core variable data dictionary and manifest were completed for determining eligibility and information required for analysis. Eligible cases provided consent and a blood sample for research studies of MM. A total of 686 MM cases were used for analyses. DNA was extracted from stored peripheral blood samples. The clinical stage was determined by chart review. Family history data were provided for 66.3% of the samples. 24.2% of those provided had a family history of MM, MGUS or lymphoma. Related samples were sparse.

Utah study:

Sampling and genetic analysis was approved by the University of Utah IRB. The study included prevalent MM cases in the state of Utah, ascertained up to 2019. Eligible cases provided consent and a blood or saliva sample from which DNA was extracted. Date of diagnosis was confirmed from chart review and Utah Cancer Registry data. Date of death was confirmed from chart review and death registry data. Other variables were sourced from chart review. A total of 430 MM cases with DNA and clinical and demographic information were available for this study. Family history data were provided for 39.8% and 10.5% of those had a family history of MM, MGUS or lymphoma. Related samples were sparse.

The Utah Population Database is supported by Huntsman Cancer Foundation, the University of Utah, and NCI grant P30 CA042014-29S9. The UCR is additionally funded by the NCI's SEER Program, HHSN261201800016I, and the US Center for Disease Control and Prevention's National Program of Cancer Registries (NU58DP007131).

The iMAGE study:

Designed to evaluate the effects of biological, chemical, physical, social and genetic influences on the risk of MM and direct comparisons by self-reported Black and White race. Approvals from the appropriate institutional review boards in accordance with the Declaration of Helsinki were obtained prior to study initiation, and informed consent was obtained from all individual participants included in the study. Eligible cases were recruited from the University of Alabama at Birmingham Hematology and Medical Oncology clinics (Birmingham, Alabama) and the Morehouse School of Medicine (Atlanta, Georgia). Patients with a diagnosis of MM were identified based on the ICD-9 classifications (203) or International Classification of Diseases for Oncology third revision code 9732/3 and confirmed based on the revised and updated International Multiple Myeloma Working Group classification criteria for MM. Each MM case was reviewed by an expert panel to ensure consistent case definitions and to minimize phenotype misclassification. Chart reviews were conducted to populate the core variables such as demographic, and clinical information required for analysis with genetic data. A total of 138 MM cases with DNA and clinical and demographic information were available for this study. Family history data were provided for 97.1% of patients. 9.0 % had a family history of MM, MGUS or lymphoma. Related samples were sparse.

DFCI study:

A total of 112 MM cases with DNA and clinical and demographic information were available for this study. Family history data were provided for 96.4% of patients. 39.8% had a family history of MM, MGUS or lymphoma. Related samples were sparse.

MD Anderson study:

MM cases were recruited from MD Anderson between 2006 and 2016. Participants provided written informed consent and peripheral blood samples for research purposes under IRB approved protocols. For a subset of the cases, response to an epidemiology questionnaire completed at time of recruitment was used to assess family history of MM, MGUS, and/or lymphoma. All other patient information was abstracted from the medical record. A total of 172 cases were included in the final analyses with family history data reported for 10.5% of the cases. Of those, 83.3% had a family history of MM, MGUS, or lymphoma. No related samples were in the study

MSKCC study:

This study is a hospital-based ascertainment that has both a familial and sporadic component. The study is approved by the institutional review board (18-339), which allows for biospecimen utilization across several other protocols related to MM cases. MM samples with a family history were collected under a protocol by the Clinical Genetics Service, where both probands and family members were ascertained where possible. Remnant blood samples were collected, and DNA was extracted using another institutional protocol that allowed de-identified research. Clinical and demographic data were obtained through a dataline query to the institutional data lake and specific clinical variables such as stage and dates of diagnoses were obtained through rigorous chart reviews. A total of 516 MSKCC cases were included in analyses; 7.8% of the cases had available family history data. Of those, 97.5% had a family history of MM, MGUS, or lymphoma. In addition, three kindreds with MM were also sequenced.

The BMTCTN study:

The BMT samples were collected through NHLBI BioLINCC for the study BMTCTN (accession # HLB00881120a). The Blood and Marrow Transplant Clinical Trials Network (BMT CTN) Protocol # 0102 trial compared progression-free survival of patients with multiple myeloma biologically assigned to receive autologous hematopoietic cell transplantation (autoHCT) followed either a second auto HCT or by allogeneic transplantation (alloHCT).  Only autologous transplant cases were used in this study. The samples were transported to MSKCC under an MTA with BioLINCC and a human subject exemption was obtained from the MSKCC IRB to use them for generating whole exome data. A total of 210 BMT cases were included in the analysis.

Next Generation Sequencing

Upon successful confirmation of the sample QC using SNP array for ancestry, sex and relatedness, Twist Human Core Exome plus custom capture was used for library preparation and enrichment. The libraries were sequenced on the Illumina NovaSeq 6000 platform. Bioinformatics analyses used BWA mem (v.07.15) to GRCh37 human genome reference and Picard 2.17.0 used for quality assurance.

Whole exome capture of each sample was performed using Twist human core exome plus CIDR custom capture panel. Libraries were prepared from 50 nanograms of genomic DNA sheared using Covaris E220 instrument and the Kappa hyper-prep kit was used to process the sheared DNA into amplified dual indexed adapter like gated fragments. Following clean up with beads, amplified libraries were pooled prior to enrichment following the twist protocol post enrichment PCR was performed using the Kappa HiFi enzyme. Libraries were sequenced on the NovaSeq 6000 platform using 100 base pair paired end runs and NovaSeq 6000 S4 reagent kit and NovaSeq XP 4-lane kit. Base calling was performed using Illumina real time analysis software. Base call files were demultiplexed from a binary format to single sample fastq files using illumina’s BCL2FASTQ demultiplexer.

Coverage metrics were calculated using DepthofCoverage walker in GATK and only those with a base call quality score of 10 or greater and reads with a mapping quality score of greater than 20 were considered. Reproducibility was estimated by using experimental blind duplicate and HapMap duplicate pairs. Concordance and missing rate among high quality genotype among pairs were evaluated for SNVs and INDELs. Pair-wise kinship coefficients were calculated using KING from the genotype data. We removed one individual of a pair until no pairs remained that were duplicates or had a 1^st^ and 2^nd^ degree relatives using the IBD sharing statistic to limit to unrelated individuals for further case-control analyses. Related samples were marked for co-segregation analyses if they carried a variant of interest. Membership in the major ancestry was calculated using principal component analysis by including HapMap and the thousand genomes populations.

Fastq files were aligned with BWA mem (Li H. 2013) version 0.7.15 to the 1000 genomes phase 2 (GRCh37) human genome reference. Duplicate molecules were flagged with Picard version 2.17.0. Base call quality score recalibration and binning (2,10,20,30) were performed using the Genome Analysis Toolkit (GATK) (McKenna et al., 2010) version v4.0.1.1. Cram files were generated using SAMTools version 1.7. GATK’s reference confidence model workflow was used to perform joint sample genotyping using GATK version 3.7. Briefly this workflow entails: 1) Producing a gVCF (genomic VCF) for each sample individually using HaplotypeCaller (--emitRefConfidence GVCF) and --max_alternate_alleles was set to 3 for all bait intervals to generate likelihoods that the sites are homozygote reference or not; 2) Joint genotyping the single sample gVCFs together with GenotypeGVCFs to produce a multi-sample VCF file.

Variant filtering was done using the Variant Quality Score Recalibration (VQSR) method (DePristo et al., 2011). For SNVs, the annotations of MQRankSum, QD, FS, ReadPosRankSum, MQ and SOR were used in the adaptive error model. HapMap3.3, Omni2.5 and 1000G phase high confidence SNP calls were used as training sites with HapMap3.3 and Omni2.5 used as the truth set. SNVs were filtered to obtain all variants up to the 99.5th percentile of truth sites (0.5% false negative rate). For indels, the annotations of FS, ReadPosRankSum, MQRankSum, QD and SOR were used in the adaptive error model (4 max Gaussians allowed). A set of curated indels obtained from the GATK resource bundle (Mills_and_1000G_gold_standard.indels.b37.vcf) were used as training and truth sites. Indels were filtered to obtain all variants up to the 99th percentile of truth sites (1% false negative rate).

An additional/optional VCF file was created where genotypes for biallelic SNPs were further refined using CalculateGenotypePosteriors using allele frequency information from 1000 genomes phase 3 data (ALL.wgs.phase3_shapeit2_mvncall_integrated_v5.20130502.sites.vcf) as well as Exome Aggregation Consortium data (ExAC.r0.3.sites.vep.vcf). (Lek, M 2016). The final multi-sample VCF files were also provided as a GRCh38 liftover by CIDR and all subsequent analysis was performed on the GRCh38 VCF file**.**

**Variant QC/QA**

Indels were normalized using the GATK toolkit’s left-align and trim function and multi-allelic variants were split into separate variant records. The VCF file was then filtered to include only variants with less than 1% allele frequency and the HapMap samples included as markers for PCA were also removed.

To select only high-quality samples, quality control (QC) filtering was performed using allele depth (AD), genotype quality (GQ), homozygous allele balance (AB), and heterozygous allele balance (ABHet). For analytical consistency, QC parameter values were made identical for the MMSEQ and UK Biobank cohorts. For each variant, sample genotypes were reverted to the reference allele (e.g. 0/0) when DP was less than 15, GQ was less than 20, AB was less than 0.9, or when ABHet was less than 0.2. If ABHet is greater than 0.8 the sample genotype field was assigned the heterozygous alternate allele (e.g. 1/1). Pairwise kinship coefficients were calculated using King. Sample pairs with second degree or closer relatedness values were found and one randomly selected sample from each related pair was removed. 3 201 cases (MMSEQ and UK Biobank) and 320 770 (UK Biobank) controls remained after QC.

**Annotation**

The variants were annotated using CAVA 2.0 (Clinical Annotation of VAriants) (23) with an hg38 reference genome and Annovar (24) using the annotation tracks: refGene, ensGene, avsnp150, cosmic70, esp6500siv2_ea, esp6500siv2_aa, esp6500siv2_all, dbnsfp33a, dbnsfp31a_interpro, exac03nontcga, exac03nonpsych, kaviar_20150923, hrcr1, exac03, nci60, dbscsnv11, gnomad_exome, gnomad_genome, revel, mcap, clinvar_20220320, gme, and icgc28.

**Pathogenicity Assertion**

Variant records were analyzed by PathoMAN. An additional level of manual curation was performed in which 100% concordance was established for variants with a review panel rating in ClinVar (RRID:SCR_006169). Splice site and missense variants that were not definitively classified by PathoMAN or previously reported in ClinVar (i.e. VUS variants) were then manually reclassified using literature and other in-silico predictions. Splice variants were classified as likely deleterious based on their SpliceAI (RRID:SCR_026278) scores when any value of donor loss, donor gain, acceptor loss, or acceptor gain exceeded 0.8. When there was no opposing ClinVar information, missense variants were classified as deleterious by their revel score (>=0.773) and CADD score (>28). These pathogenic and deleterious variants (qualifying variants) are found in the **supplementary data file**.

UK Biobank

UK Biobank (UKB) is a large-scale biomedical database and research resource containing deidentified, genetic, lifestyle and health information from half a million UK participants. UK Biobank’s database - which includes blood samples, heart and brain scans and genetic data of the 500,000 volunteer participants - is globally accessible to approved researchers who are undertaking health-related research in the public interest.

UKB recruited 500,000 people aged between 40-69 years in 2006-2010 from across the UK. With their consent, they provided detailed information about their lifestyle and physical measures and had blood, urine and saliva samples collected and stored for future analysis. Access to UK Biobank for this study was approved through project 50092. UK biobank whole exome data was generated as detailed in Szustakowski et al. (2021).

**UKB Cohort Selection**

Multiple myeloma cases were selected from UKB based on ICD10 codes found in the cancer registry: type of cancer and histology fields. MM cases were divided into incident and prevalent cancer status by comparing age at enrollment to age at cancer diagnosis. Non-cancer controls were selected by excluding participants who had any cancer summary diagnoses, cancer related cause of death, or any code listed for self-reported cancer or type of cancer in the cancer registry. We also removed any individuals from controls who had a reported history of MGUS.

For both case and control cohorts, individuals whose self-reported sex did not match their genetically derived sex were removed as were those who opted out of the study. Individuals were only included if they had data for age at recruitment, sex, and genetic principal components and if the participant was of predominantly European self-reported ancestry **(Supplemental Table 2).** If individuals were related as evidenced by their genetic relatedness coefficient calculated using King (RRID:SCR_009251), one of the individuals from the pair was removed. Among the UKB cases and controls, individuals who had 10 or more closely related (3rd degree or closer) relatives in the data were removed.

**Comparison of AFs of common variants in MMSEQ and UKB cohorts**

To ensure the MMSEQ and UKB datasets were comparable, we performed an association test on variants common to both MMSEQ and UK Biobank WES data using Plink 1.9 (RRID:SCR_001757). We filtered for variants with allele frequencies between 5% and 95%. QC was performed as previously stated in QC section and the resulting merged VCF file of cases and controls contained 324 214 samples and 554 variant records shared among MMSEQ and UKB. We performed an association test using PLINK with genomic control. The resulting QQ-plot showed there was negligible genomic inflation with λ = 0.99, suggesting that both datasets were comparable and were suitable for a case control analysis of exonic rare variants (**Supplemental Figure 3**).

TP53 bam inspection

In addition to the allele balance threshold in the QC step, we visually inspected the bam files of samples with TP53 mutations looking for evidence of clonal hematopoiesis, somatic mosaicism or simply a very low variant allele frequencies (VAF>35%), to exclude them from the analysis. This inspection was performed manually using IGV to verify that variants were not clonal somatic events and included analyzing allele balance, depth of coverage, strand bias and regional analyses for copy number events. All pathogenic variants were verified to be bona fide variants that passed stringent thresholds for germline variations.

Survival Analysis

Among the 7 centers, there were 1 306 / 2 937 patients with death records available for the OS data. Since only 4 genes showed mutations in greater than 3% of cases, we limited OS analyses to just those genes (*TP53, CHEK2, DIS3, and ATM*). OS was defined as time from diagnosis to death or last follow-up. For patients with only the year of diagnosis or death available we assumed the date to be the first of the January of that year. For 3 cohorts, Utah, MDA and UAB, we imputed the last year of follow-up to be the last year of death in that cohort. We could not obtain OS for BMT-CTN cohort since we had no follow-up information.

**Supplementary Table 1**: MMSEQ demographics of samples across centers

|  | **Overall (N=2,264)** | | **BMT (N=210)** | | **DFCI (N=112)** | | **MAYO (N=686)** | | **MDA (N=172)** | | **MSKCC (N=516)** | | **UAB (N=138)** | | **Utah (N=430)** | |
| --- | --- | --- | --- | --- | --- | --- | --- | --- | --- | --- | --- | --- | --- | --- | --- | --- |
| **Age** |  |  |  |  |  |  |  |  |  |  |  |  |  |  |  |  |
| <40 | 102 | 4.5% | 13 | 6.2% | 14 | 12.5% | 22 | 3.2% | 11 | 6.4% | 20 | 3.9% | 6 | 4.3% | 16 | 3.7% |
| 40-45 | 153 | 6.8% | 27 | 12.9% | 9 | 8.0% | 44 | 6.4% | 13 | 7.6% | 33 | 6.4% | 15 | 10.9% | 12 | 2.8% |
| 46-50 | 321 | 14.2% | 35 | 16.7% | 28 | 25.0% | 88 | 12.8% | 31 | 18.0% | 67 | 13.0% | 19 | 13.8% | 53 | 12.3% |
| 51-55 | 481 | 21.2% | 51 | 24.3% | 20 | 17.9% | 122 | 17.8% | 52 | 30.2% | 116 | 22.5% | 41 | 29.7% | 79 | 18.4% |
| 56-60 | 596 | 26.3% | 56 | 26.7% | 18 | 16.1% | 159 | 23.2% | 49 | 28.5% | 155 | 30.0% | 56 | 40.6% | 103 | 24.0% |
| 61-65 | 502 | 22.2% | 28 | 13.3% | 18 | 16.1% | 182 | 26.5% | 13 | 7.6% | 125 | 24.2% | 0 | 0.0% | 136 | 31.6% |
| >65 | 109 | 4.8% | 0 | 0.0% | 5 | 4.5% | 69 | 10.1% | 3 | 1.7% | 0 | 0.0% | 1 | 0.7% | 31 | 7.2% |
| **Sex** |  |  |  |  |  |  |  |  |  |  |  |  |  |  |  |  |
| Female | 910 | 40.2% | 77 | 36.7% | 56 | 50.0% | 294 | 42.9% | 59 | 34.3% | 202 | 39.1% | 54 | 39.1% | 168 | 39.1% |
| Male | 1354 | 59.8% | 133 | 63.3% | 56 | 50.0% | 392 | 57.1% | 113 | 65.7% | 314 | 60.9% | 84 | 60.9% | 262 | 60.9% |
| **Ethnicity** |  |  |  |  |  |  |  |  |  |  |  |  |  |  |  |  |
| European | 2210 | 97.6% | 210 | 100.0% | 112 | 100.0% | 650 | 94.8% | 171 | 99.4% | 516 | 100.0% | 138 | 100.0% | 413 | 96.0% |
| African American | 5 | 0.2% | 0 | 0.0% | 0 | 0.0% | 2 | 0.3% | 1 | 0.6% | 0 | 0.0% | 0 | 0.0% | 2 | 0.5% |
| East Asian | 4 | 0.2% | 0 | 0.0% | 0 | 0.0% | 1 | 0.1% | 0 | 0.0% | 0 | 0.0% | 0 | 0.0% | 3 | 0.7% |
| Native American | 4 | 0.2% | 0 | 0.0% | 0 | 0.0% | 2 | 0.3% | 0 | 0.0% | 0 | 0.0% | 0 | 0.0% | 2 | 0.5% |
| Hispanic | 2 | 0.1% | 0 | 0.0% | 0 | 0.0% | 2 | 0.3% | 0 | 0.0% | 0 | 0.0% | 0 | 0.0% | 0 | 0.0% |
| Other | 2 | 0.1% | 0 | 0.0% | 0 | 0.0% | 0 | 0.0% | 0 | 0.0% | 0 | 0.0% | 0 | 0.0% | 2 | 0.5% |
| Unknown | 37 | 1.6% | 0 | 0.0% | 0 | 0.0% | 0 | 0.0% | 0 | 0.0% | 0 | 0.0% | 0 | 0.0% | 0 | 0.0% |
| **Stage (ISS)** |  |  |  |  |  |  |  |  |  |  |  |  |  |  |  |  |
| 1 | 500 | 22.1% | 0 | 0.0% | 31 | 27.7% | 161 | 23.5% | 2 | 1.2% | 218 | 42.2% | 36 | 26.1% | 52 | 12.1% |
| 2 | 453 | 20.0% | 57 | 27.1% | 15 | 13.4% | 165 | 24.1% | 1 | 0.6% | 117 | 22.7% | 41 | 29.7% | 57 | 13.3% |
| 3 | 403 | 17.8% | 153 | 72.9% | 13 | 11.6% | 97 | 14.1% | 6 | 3.5% | 73 | 14.1% | 20 | 14.5% | 41 | 9.5% |
| Unknown | 908 | 40.1% | 0 | 0.0% | 53 | 47.3% | 263 | 38.3% | 163 | 94.8% | 108 | 20.9% | 41 | 29.7% | 280 | 65.1% |
| **Family History (MM, MGUS or Lymphoma)** | | | |  |  |  |  |  |  |  |  |  |  |  |  |  |
| Yes | 237 | 10.5% | 0 | 0.0% | 43 | 38.4% | 110 | 16.0% | 15 | 8.7% | 39 | 7.6% | 12 | 8.7% | 18 | 4.2% |
| No | 689 | 30.4% | 0 | 0.0% | 65 | 58.0% | 345 | 50.3% | 3 | 1.7% | 1 | 0.2% | 122 | 88.4% | 153 | 35.6% |
| Unknown | 1338 | 59.1% | 210 | 100.0% | 4 | 3.6% | 231 | 33.7% | 154 | 89.5% | 476 | 92.2% | 4 | 2.9% | 259 | 60.2% |
| **Vital Status** |  |  |  |  |  |  |  |  |  |  |  |  |  |  |  |  |
| Alive | 1244 | 54.9% | 149 | 71.0% | 0 | 0.0% | 192 | 28.0% | 87 | 50.6% | 392 | 76.0% | 103 | 74.6% | 321 | 74.7% |
| Deceased | 903 | 39.9% | 61 | 29.0% | 0 | 0.0% | 489 | 71.3% | 85 | 49.4% | 124 | 24.0% | 35 | 25.4% | 109 | 25.3% |
| Lost to follow-up | 5 | 0.2% | 0 | 0.0% | 0 | 0.0% | 5 | 0.7% | 0 | 0.0% | 0 | 0.0% | 0 | 0.0% | 0 | 0.0% |
| Unknown | 112 | 4.9% | 0 | 0.0% | 112 | 100.0% | 0 | 0.0% | 0 | 0.0% | 0 | 0.0% | 0 | 0.0% | 0 | 0.0% |

**Supplemental Table 1: MMSEQ demographics across centers.** Demographic variables from MM cases gathered from 6 compiled datasets. These patients were selected for early onset MM and a family history of cancer (MM, MGUS, Lymphoma).

**Supplementary Table 2**: UKBB demographics and carrier counts for MM cases and non-cancer controls

**Characteristic** **Overall** N=321,707 **Cases** N=937 **Controls** N=320,770

**Age** Combined Ages Age at Diagnosis Age at Recruitment

<40 13 <0.1% 8 0.9% 5 <0.1%

40-45 37529 11.7% 18 1.9% 37511 11.7%

46-50 48077 14.9% 39 4.2% 48038 15.0%

51-55 53973 16.8% 86 9.2% 53887 16.8%

56-60 63654 19.8% 103 11.0% 63551 19.8%

61-65 70278 21.8% 174 18.6% 70104 21.9%

>65 39080 12.1% 510 54.4% 38570 12.0%

**Sex**

Female 174167 54.0% 388 41.4% 173779 54.2%

Male 147541 45.7% 550 58.7% 146991 45.8%

**Carriers**

*ATM* 457 0.14% 4 0.43% 453 0.14%

*CHEK2* 2265 0.70% 15 1.60% 2250 0.70%

*BRCA2* 578 0.18% 4 0.43% 574 0.18%

*DIS3* 708 0.22% 2 0.21% 706 0.22%

*BRCA1* 139 0.04% 1 0.11% 138 0.04%

*USP45* 429 0.13% 0 0.00% 429 0.13%

*TP53* 39 0.01% 1 0.11% 38 0.01%

*KDM1A* 144 0.04% 0 0.00% 144 0.04%

*ARID1A* 8 <0.01% 0 0.00% 8 <0.01%

None 316937 98.5% 911 97.2% 316026 98.5%

**Vital Status**

Alive 309727 96.3% 508 54.2% 309219 96.4%

Deceased 11581 3.6% 30 3.2% 11551 3.6%

**Supplementary Table 2: UKBB demographics and carrier counts for MM cases and non-cancer controls.** Demographic data from UKBB carriers and non-carriers in cases and controls.

**Supplemental Table 3: Cytogenetic abnormalities among MSKCC MM patients**

| **Status** | **Carriers** | **Non-Carriers** | **OR** | **95% CI** | **P** |
| --- | --- | --- | --- | --- | --- |
| t(4;14) | 1 | 27 | 0.53 | 0.01-3.45 | 1.00 |
| t(11;14) | 5 | 63 | 1.20 | 0.34-3.34 | 0.79 |
| Loss of TP53 | 2 | 55 | 0.50 | 0.06-2.08 | 0.56 |
| Total abnormalities | 8 | 130 | 0.87 | 0.33-2.09 | 0.84 |

**Supplemental Table 3: Cytogenetic abnormalities among MSKCC MM patients**. Cytogenetic data were collected from MSKCC multiple myeloma patients with counts cytogenetic abnormalities are denoted in the table.

**Supplementary Table 4 :** Age at Onset for MM mutation carriers compared to WT

| **GENE** | **Counts** | **Mean Age** | **Median Age** | **Min Age** | **Max Age** | **P-value** |
| --- | --- | --- | --- | --- | --- | --- |
| *CHEK2* | 85 | 57 | 58 | 28 | 74 | 0.26 |
| *TP53* | 17 | 57.5 | 59 | 37 | 67 | 0.92 |
| *ATM* | 16 | 58.9 | 57.1 | 50.8 | 75.6 | 0.89 |
| *KDM1A* | 7 | 51.4 | 49 | 45 | 59 | **0.026** |
| *DIS3* | 11 | 58.5 | 63 | 41 | 76.3 | 0.75 |
| *BRCA2* | 9 | 61.1 | 62 | 49 | 70.4 | 0.37 |
| *BRCA1* | 4 | 59.1 | 58.6 | 58 | 61 | 0.9 |
| *ARID1A* | 3 | 58.7 | 62 | 44 | 70 | 0.86 |
| *USP45* | 1 | 43 | 43 | 43 | 43 | 0.13 |
| Non-Carriers | 3052 | 58.5 | 59 | 20 | 88 | - |

**Supplemental Table 4: Age at diagnosis for carriers vs non-carriers.** The table shows the average age at diagnosis for carriers of a pathogenic or deleterious variant in a certain gene among MM (MMSEQ and UKBB) cases. The Wilcoxon ranked-sum test compares the ages of carriers vs non-carriers of the gene in question. The only gene that showed a significantly different distribution of ages at diagnosis when compared to non-carriers was KDM1A.**Supplemental Table 5: MM cases from the TP53 database**

| Family_ID | 522 | 552 | 71 |
| --- | --- | --- | --- |
| Family_code | PRO08 | BOU08-C | Law91-STS170(SARC36) |
| Class | LFL | LFL | LFS |
| Generations_analyzed | 5 | 4 | 4 |
| Germline_mutation | TP53 | TP53 | TP53 |
| Country | Czech Republic | France | USA |
| Population | Eastern Europe | Western Europe | Northern America |
| Region | Europe | Europe | Americas |
| hg19_Chr17_coordinates | 7577539 | 7579312 | 7578532 |
| hg38_Chr17_coordinates | 7674221 | 7675994 | 7675214 |
| ExonIntron | 7-exon | 4-exon | 5-exon |
| Codon_number | 248 | 125 | 133 |
| Description | C>T | G>A | T>C |
| c_description | c.742C>T | c.375G>A | c.398T>C |
| g_description | g.7577539G>A | g.7579312C>T | g.7578532A>G |
| g_description_GRCh38 | g.7674221G>A | g.7675994C>T | g.7675214A>G |
| Type | G:C>A:T at CpG | G:C>A:T at CpG | A:T>G:C |
| WT_nucleotide | C | G | T |
| Splice_site | no | no | no |
| WT_AA | Arg | Thr | Met |
| Mutant_AA | Trp | Thr | Thr |
| Effect | missense | splice | missense |
| AGVGDClass | C65 | NA | C45 |
| TransactivationClass | non-functional | NA | non-functional |
| DNE_LOFclass | DNE_LOF | notDNE_notLOF | DNE_LOF |
| DNEclass | Yes | NA | No |
| ProtDescription | p.R248W | p.T125T | p.M133T |
| COSMIClink | 10656 | 43904 | 43723 |
| CLINVARlink | 12347 | 177825 | 12357 |
| Hotspot | yes | yes | yes |
| FamilyCase | paternal grandmother | paternal aunt | NA |
| FamilyCase_group | 2nd degree relative | 2nd degree relative | NA |
| Sex | F | F | M |
| Dead | TRUE | TRUE | TRUE |
| Unaffected | FALSE | FALSE | FALSE |
| Morphology | Multiple myeloma(C42.1) | Multiple myeloma(C42.1) | Multiple myeloma (C42.1) |
| Age_at_diagnosis | 73 | 68 | 45 |
| PubMed | 18068537 | 18511570 | 1933902 |
| Morphogroup | PLASMA CELL TUMORS | PLASMA CELL TUMORS | PLASMA CELL TUMORS |

**Supplemental Table 5: MM cases from the *TP53* database**. These data were sourced from https://tp53.isb-cgc.org/search_germline_mut.

**Supplemental Table 6: Comparison of prior MM rare variant studies**

| **Study Name** | **# Samples** | **Study Design** | **Significant Genes** | **Year** | **PMID** |
| --- | --- | --- | --- | --- | --- |
| Wei *et al* | 50 Probands  400 early onset cases | Family history enriched | *KDM1A* | 2019 | [29559475](https://pubmed.ncbi.nlm.nih.gov/29559475/) |
| Pertesi *et al* | 66 Familial Cases, 148 Cases | Familial Study | *DIS3* | 2019 | [30967618](https://pubmed.ncbi.nlm.nih.gov/30967618/) |
| Catalano *et al* | 21 Families, 46 affected and 20 unaffected | Familial Study, genes under GWAS peaks | - | 2021 | 33583942 |
| Thibaud *et al* | 895 sporadic cases from MMRF, 786 ISMMS | RVAS with case and controls | *BRCA1/2* | 2024 | 39283238 |
| Rodrigues *et al* | 954 sporadic cases from MMRF, 99 familial cases | RVAS with case and controls (gnomAD) | - | 2024 | 39845416 |
| Boddicker *et al* | 2,138 Cases | RVAS with case and controls (MAYO) | *TP53, CHEK2* | 2025 | 40253392 |
| Conry *et al* | 3,201 Cases | RVAS with case (MMSEQ/UKB) and controls (UKB) | *TP53, ATM, CHEK2, ARID1A, KDM1A* | 2025 | **-** |

**Supplemental Table 6: Comparison of prior MM rare variant studies.** Comparison of recent MM study sizes, methods, and significantly associated genes.


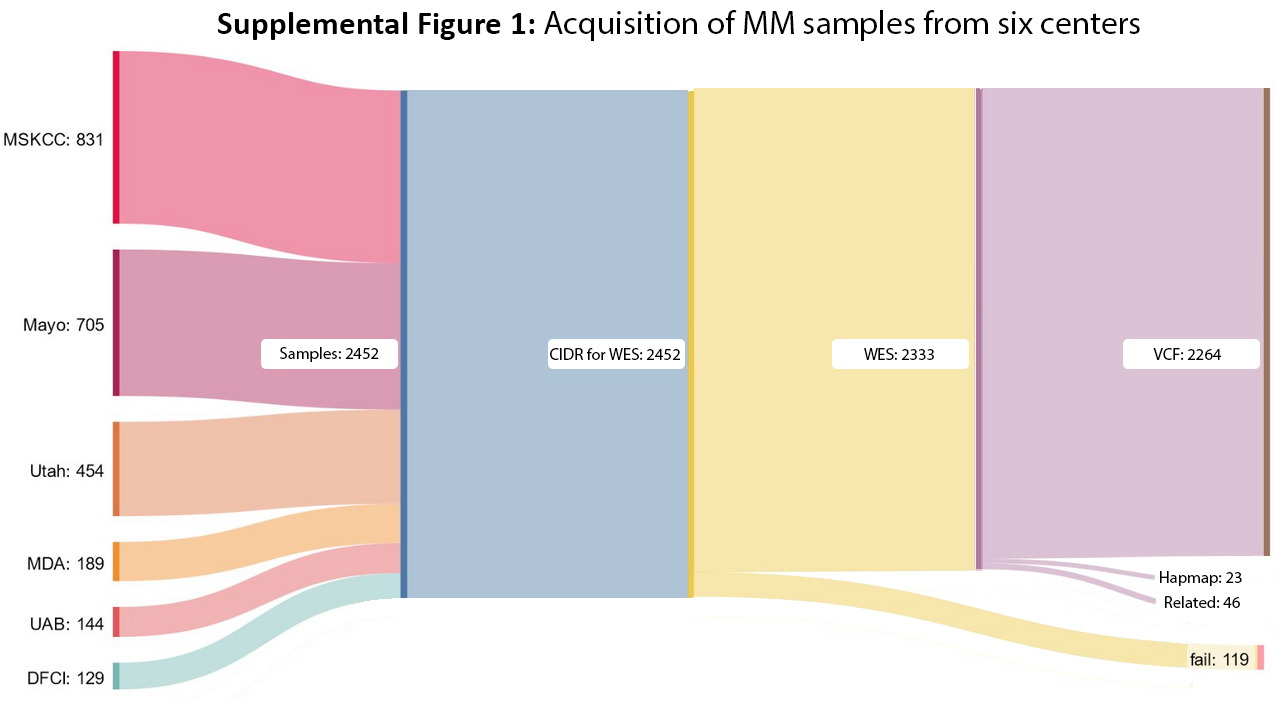


**Supplemental Figure 1: Acquisition of multiple myeloma cases and clinical data from six centers**. The samples were sequenced by CIDR with some samples removed owing to failed QC.


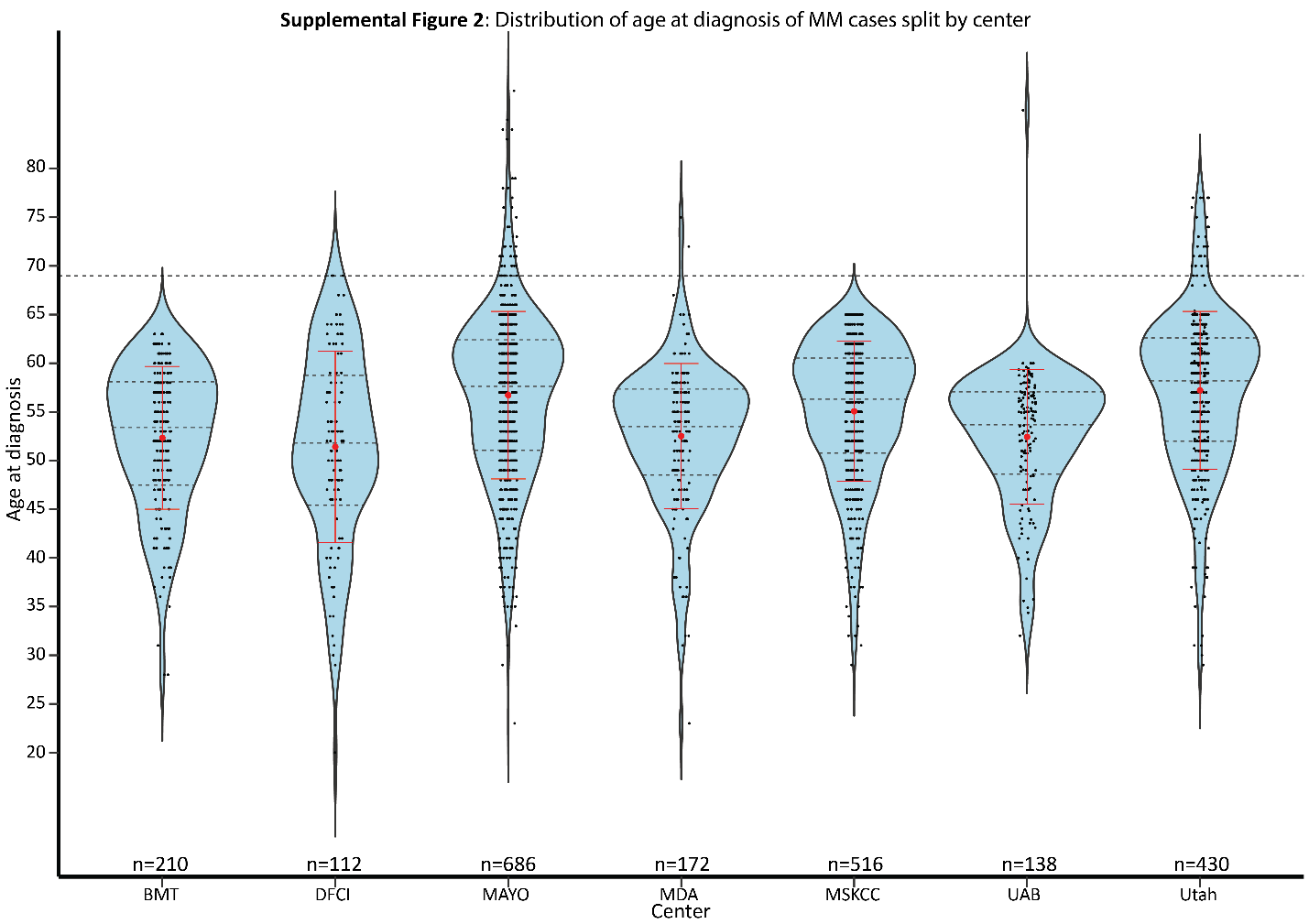


**Supplemental Figure 2: Age at diagnosis from each center.** The age at diagnosis for each center is distributed younger than the average age of diagnosis for multiple myeloma. The horizontal line denotes the average age at onset (SEER) of multiple myeloma.


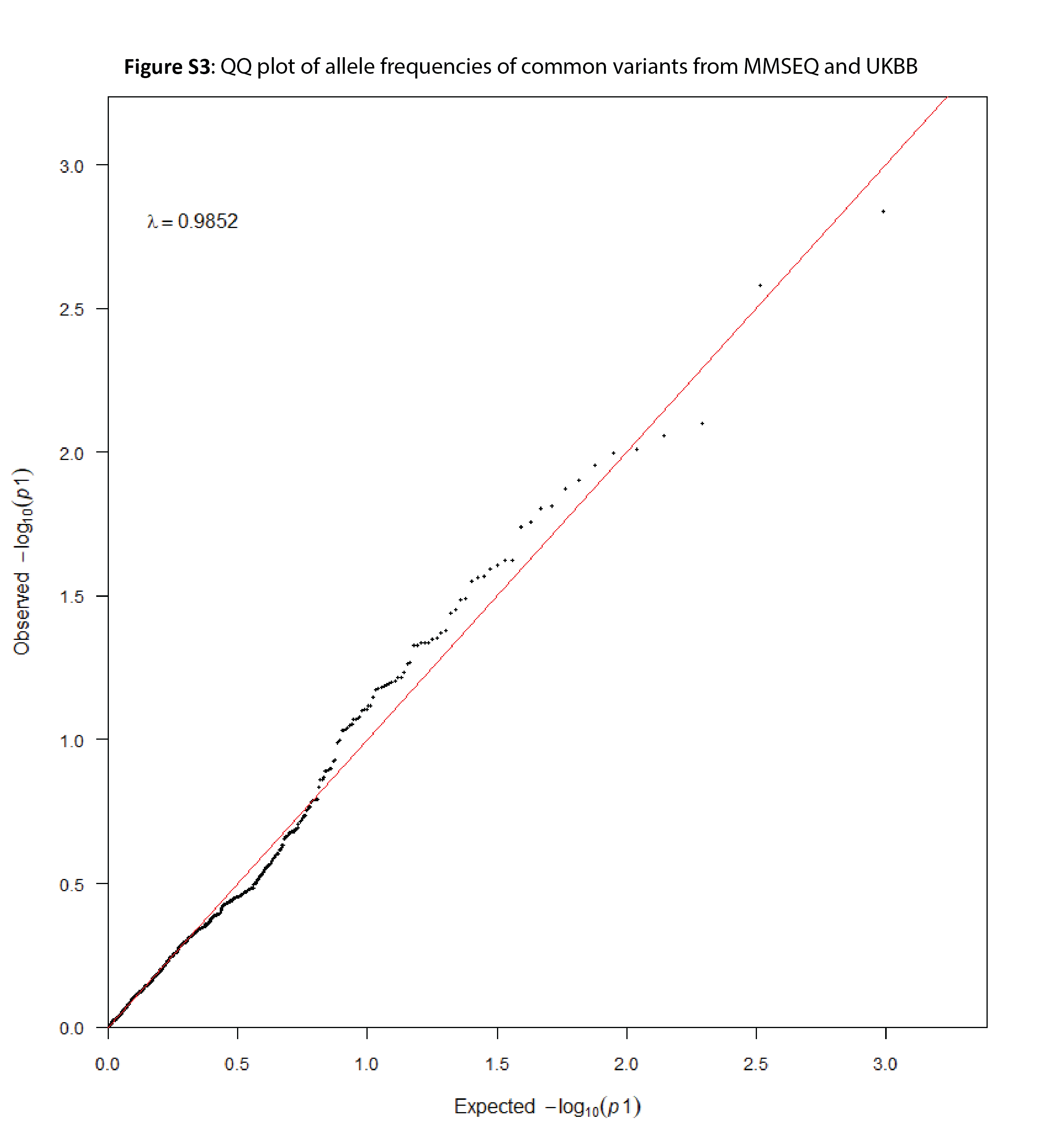
**Supplemental Figure 3: QQ plot of common variants in MMSEQ and UKBB dataset.** The QQ plot shows the close association of the rank ordered allele frequencies for common variants. The lambda value is very close to 1 suggesting that the common variants are drawn from the same distribution.


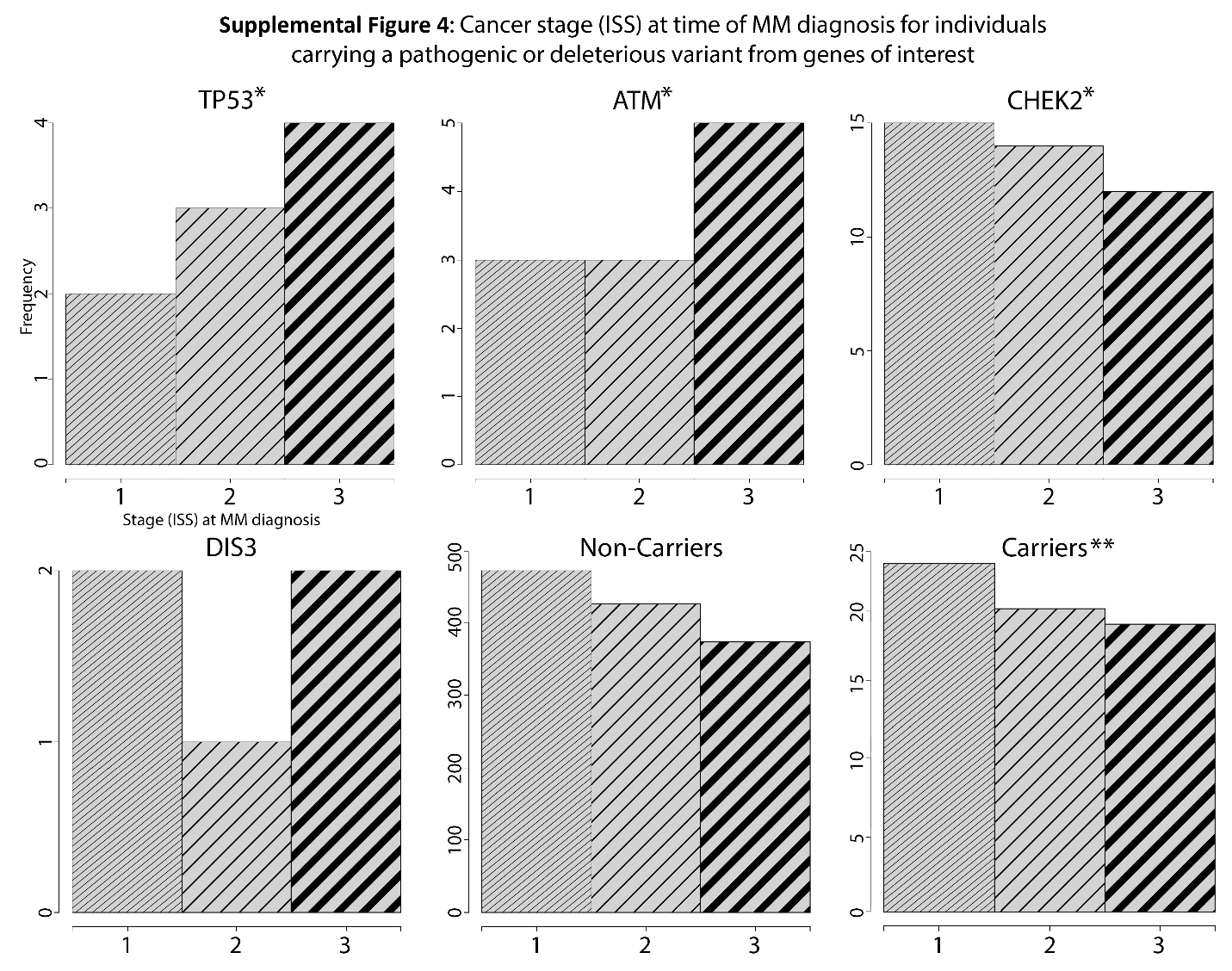


**Supplemental Figure 4: Distribution of ISS-Stage for MM by carrier gene.** In the histograms, the x-axis denotes ISS stage at diagnosis and the y-axis shows the counts of carriers of pathogenic variants for each ISS stage. Some individuals did not have Stage information available and were not included in the calculation. Genes with significant associations from gene burden test are indicated with a *. Combined carriers from TP53, CHEK2, ATM, KDM1A, and ARID1A are indicated with **.


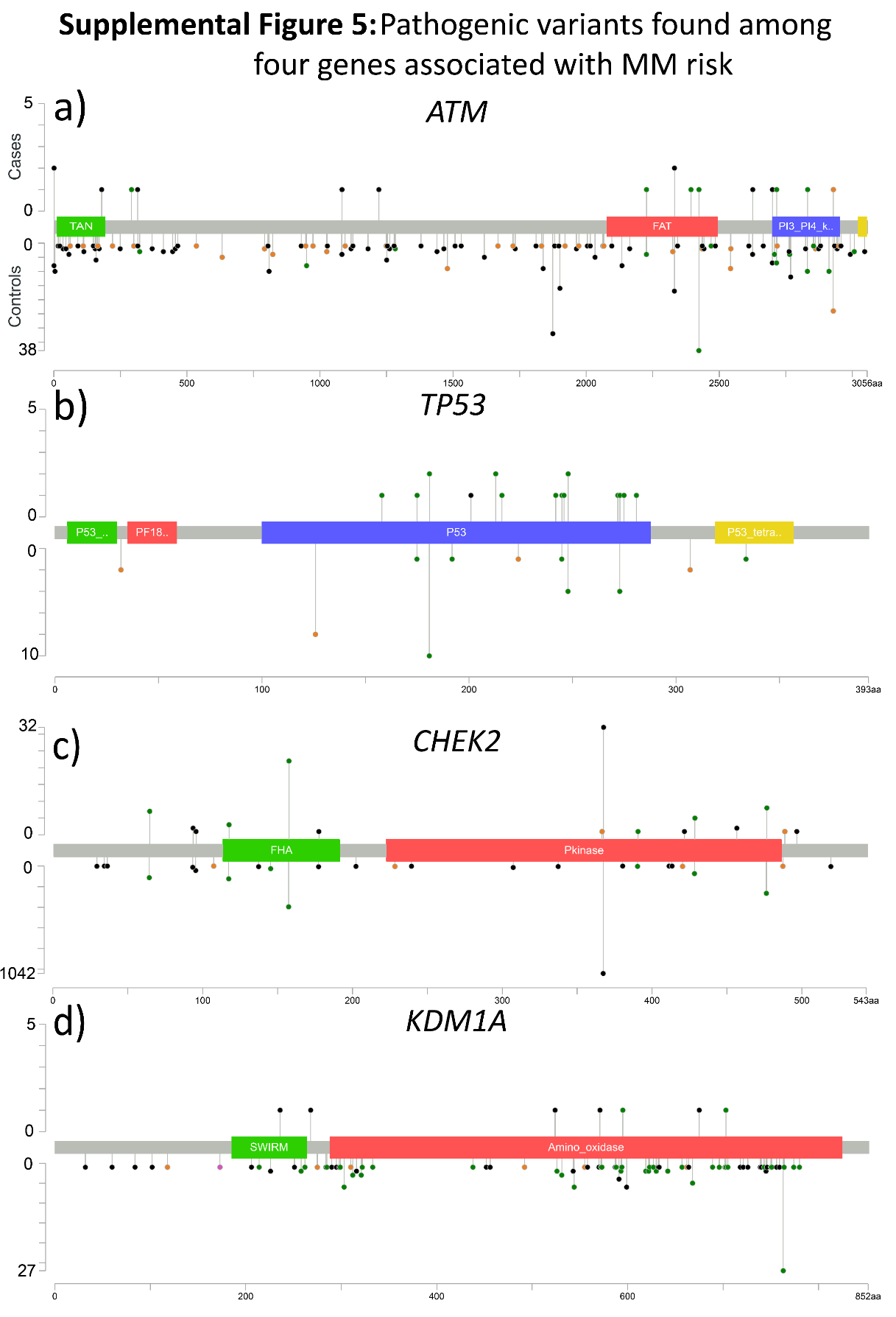


**Supplemental Figure 5: Pathogenic variants from four genes significantly associated with MM risk.** The y-axis in each plot shows the allele count for each variant in cases (top) and controls (bottom). The x-axis shows each variant’s position in the gene measured by amino acid position. ARID1A is not shown owing to low variant counts.


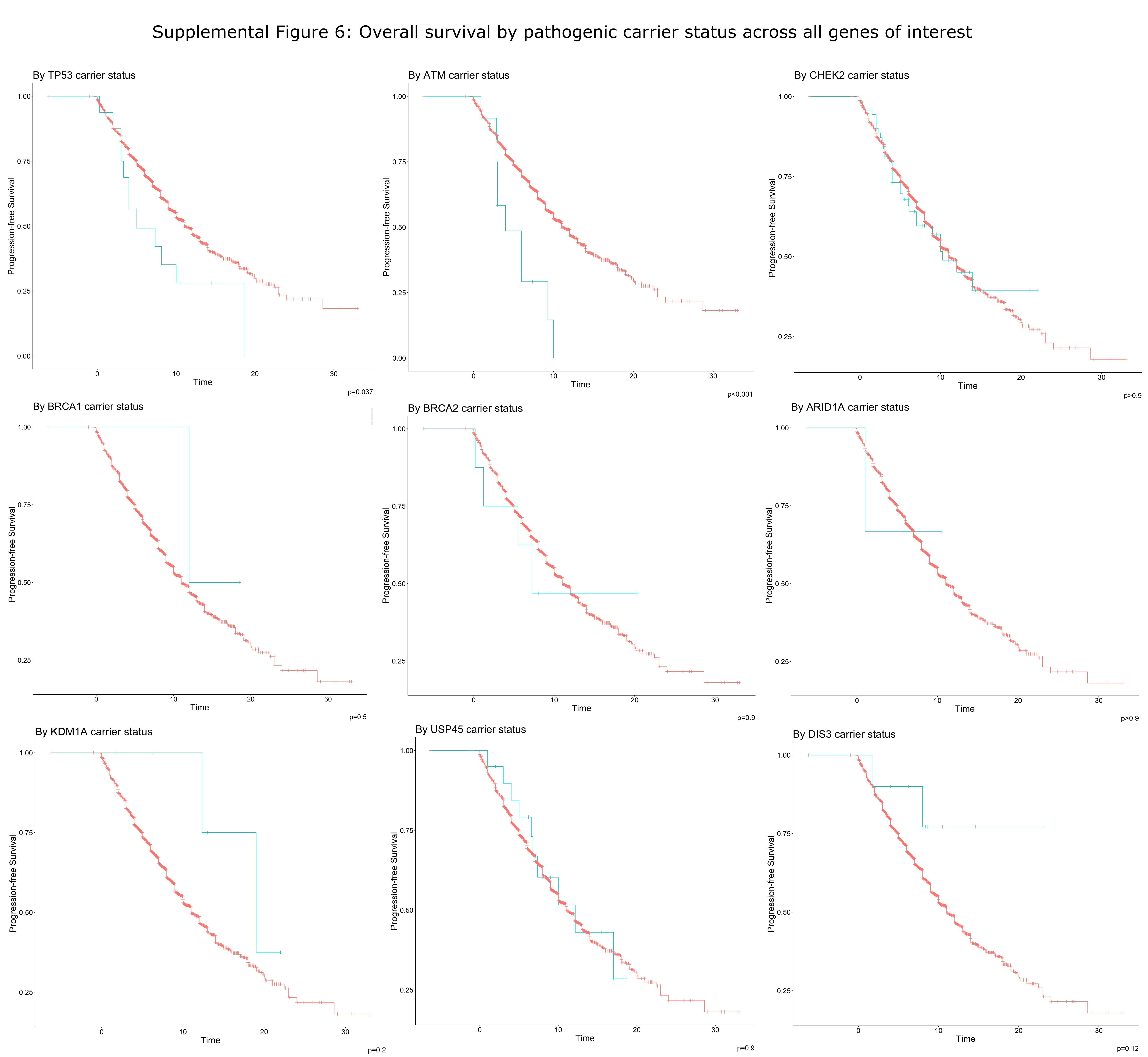


**Supplemental Figure 6: Overall survival by pathogenic carrier status across all genes of interest.**
